# (2,3-Difluoro­phen­yl)(4-tosyl­piperazin-1-yl)methanone

**DOI:** 10.1107/S1600536812051690

**Published:** 2013-01-04

**Authors:** S. Sreenivasa, K. E. ManojKumar, P. A. Suchetan, J. Tonannavar, Yashshwita Chavan, B. S. Palakshamurthy

**Affiliations:** a Department of Studies and Research in Chemistry, Tumkur University, Tumkur, Karnataka 572 103, India; b Department of Studies and Research in Chemistry, U.C.S., Tumkur University, Tumkur, Karnataka 572 103, India; c Department of Physics, Karnatak University, Dharwad, Karnataka 580 003, India; d Department of Studies and Research in Physics, U.C.S., Tumkur University, Tumkur, Karnataka 572 103, India

## Abstract

In the title compound, C_18_H_18_F_2_N_2_O_3_S, the piperazine ring adopts a chair conformation. The dihedral angle between the sulfonyl-bound benzene ring and the best fit plane throught the six non-H atoms of the piperazine ring is 69.4 (2)°, while those between the fluoro­benzene and sulfonyl rings and the fluoro­benzene and piperazine rings are 30.97 (2) and 75.98 (2)°, respectively. In the crystal, mol­ecules are connected to form a tetra­meric unit through C—H⋯O hydrogen bonds. The structure is further stabilized by weak inter­molecular C—H⋯F inter­actions, generating *C*(8) and *C*(7) chains running along [100].

## Related literature

For the synthesis, characterization and biological activity of piperazine and its derivatives, see: Gan *et al.* (2009*a* ▶,*b* ▶). For hydrogen-bond motifs, see: Bernstein *et al.* (1995 ▶).
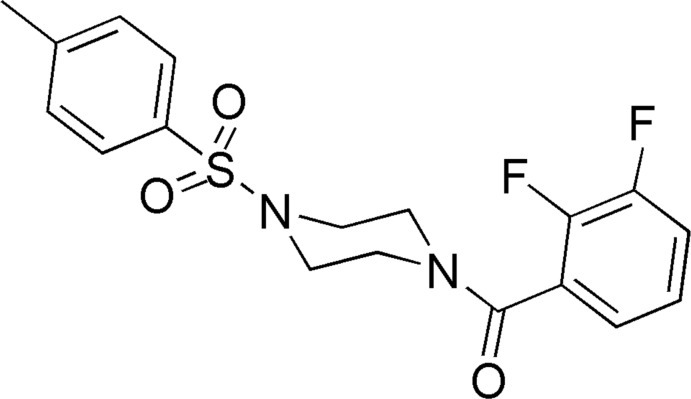


## Experimental

### 

#### Crystal data

C_18_H_18_F_2_N_2_O_3_S
*M* *_r_* = 380.40
Monoclinic, 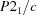

*a* = 17.0456 (4) Å
*b* = 7.6026 (1) Å
*c* = 15.5113 (3) Å
β = 113.513 (1)°
*V* = 1843.22 (6) Å^3^
*Z* = 4
Mo *K*α radiation
μ = 0.22 mm^−1^
*T* = 298 K
0.28 × 0.26 × 0.20 mm

#### Data collection

Bruker APEXII CCD diffractometer
Absorption correction: multi-scan (*SADABS*; Bruker, 2009 ▶) *T*_min_ = 0.942, *T*_max_ = 0.958
11988 measured reflections
2359 independent reflections
1930 reflections with *I* > 2σ(*I*)
*R*_int_ = 0.023
θ_max_ = 22.4°

#### Refinement

*R*[*F* ^2^ > 2σ(*F* ^2^)] = 0.033
*wR*(*F* ^2^) = 0.092
*S* = 1.06
2359 reflections
236 parameters
H-atom parameters constrained
Δρ_max_ = 0.15 e Å^−3^
Δρ_min_ = −0.23 e Å^−3^

### 

Data collection: *APEX2* (Bruker, 2009 ▶); cell refinement: *APEX2* and *SAINT-Plus* (Bruker, 2009 ▶); data reduction: *SAINT-Plus* and *XPREP* (Bruker,2009 ▶); program(s) used to solve structure: *SHELXS97* (Sheldrick, 2008 ▶); program(s) used to refine structure: *SHELXL97* (Sheldrick, 2008 ▶); molecular graphics: *ORTEP-3* (Farrugia, 2012 ▶); software used to prepare material for publication: *SHELXL97*.

## Supplementary Material

Crystal structure: contains datablock(s) I, global. DOI: 10.1107/S1600536812051690/sj5290sup1.cif

Structure factors: contains datablock(s) I. DOI: 10.1107/S1600536812051690/sj5290Isup2.hkl

Supplementary material file. DOI: 10.1107/S1600536812051690/sj5290Isup3.cml

Additional supplementary materials:  crystallographic information; 3D view; checkCIF report

## Figures and Tables

**Table 1:** Hydrogen-bond geometry (Å, °)

*D*—H⋯*A*	*D*—H	H⋯*A*	*D*⋯*A*	*D*—H⋯*A*
C4—H4⋯O3^i^	0.93	2.58	3.495 (3)	169
C8—H8*A*⋯O3^ii^	0.97	2.34	3.255 (3)	157
C10—H10*B*⋯O1^iii^	0.97	2.48	3.402 (3)	159
C8—H8*B*⋯F1^iv^	0.97	2.57	3.518 (3)	165
C11—H11*A*⋯F2^iv^	0.97	2.56	3.296 (3)	133

Symmetry codes: (i) 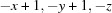; (ii) 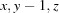; (iii) 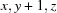; (iv) 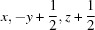.

**Fig. 1.**
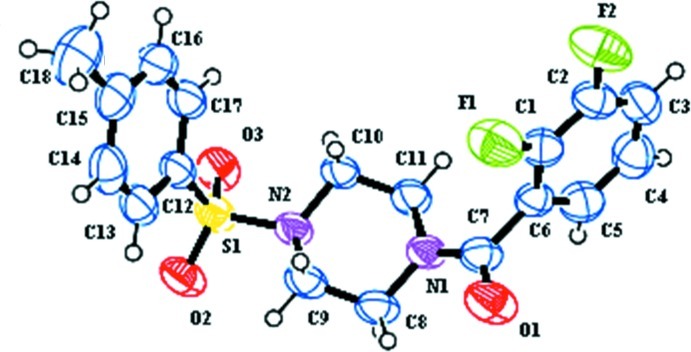
Molecular structure of the title compound, showing the atom-labeling scheme. Displacement ellipsoids are drawn at the 50% probability level.

**Fig. 2.**
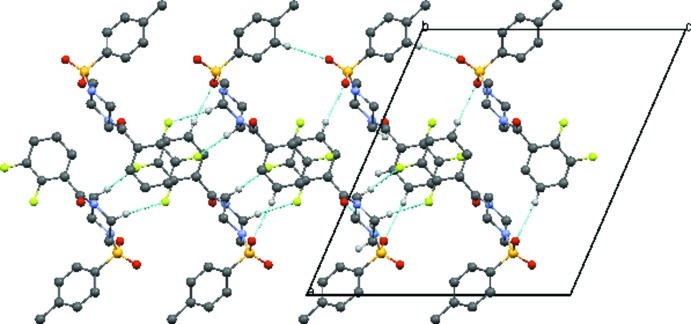
Molecular packing in the title compound. Hydrogen bonds are shown as dashed lines.

## supplementary crystallographic information

### Comment

Numerous piperazine derivatives such as aryl amides, sulphonamides, Mannich bases, Schiff's bases, thiazolidinones, azetidinones, imidazolinones have shown a wide spectrum of biological activities *viz*. anti-inflammatory, antibacterial, antimalarial, anticonvulsant, antipyretic, antitumor, anthelmintics, analgesic, antidepressant, antifungal, antitubercular, anticancer, antidiabetic effects (Gan *et al.*, 2009*a*, 2009*b*). Keeping this in mind, we synthesized the title compound to study its crystal structure.

The compound crystallizes in monoclinic crystal system and the space group *P*2_1_/*c*. In the crystal structure, the piperazine ring adopts chair conformation. The dihedral angle between the sulfonyl bound benzene ring and the best fit plane through all six atoms of the piperazine ring is 69.4 (2)°, while those between the fluorobenzene and the sulfonyl rings and the fluorobenzene and the piperazine rings are 30.97 (2)° and 75.98 (2)° respectively. In the crystal structure molecules form a tetrameric unit generating alternate *R*_2_^2^(22), (C8—H8A···O3, C10—H10B···O1) and inversion related C4—H4···O3 *R*_2_^2^(10) rings (Bernstein *et al.*1995). The structure is further stabilized by weak intermolecular C11—H11A···F2 and C8—H8B···F1 interactions.

### Experimental

1-Tosylpiperazine (0.01 mmol) and triethylamine (0.02 mmol) were dissolved in 10 ml of dichloromethane (CH_2_Cl_2_). The mixture was cooled to 0°C and 1-propanephosphonic acid anhydride (0.02 mmol) and 2,4-diflurobenzoic acid (0.01 mmol) added. The reaction mixture was stirred at 80°C for 14 h. The reaction was monitored by TLC and the solvent removed yielding the crude product. The crude mass was purified by chromatography on 230–400 silica gel with petroleum ether and ethyl acetate as eluents. Single crystals of the title compound were obtained from slow evaporation of a solution of the compound in petroleum ether and ethyl acetate (1:4).

### Refinement

H atoms were positioned with idealized geometry using a riding model with C—H = 0.93 - 0.97 Å. The isotropic displacement parameters for all H atoms were set to 1.2 times *U*_eq_ of the parent atom or 1.5 times that of the parent atom for CH_3_.

### Crystal data

**Table:**

C_18_H_18_F_2_N_2_O_3_S	Prism
*M**_r_* = 380.40	*D*_x_ = 1.371 Mg m^−^^3^
Monoclinic, *P*2_1_/*c*	Melting point: 457 K
Hall symbol: -P 2ybc	Mo *K*α radiation, λ = 0.71073 Å
*a* = 17.0456 (4) Å	Cell parameters from 2364 reflections
*b* = 7.6026 (1) Å	θ = 2.6–22.4°
*c* = 15.5113 (3) Å	µ = 0.22 mm^−^^1^
β = 113.513 (1)°	*T* = 298 K
*V* = 1843.22 (6) Å^3^	Prism, colourless
*Z* = 4	0.28 × 0.26 × 0.20 mm
*F*(000) = 792	

### Data collection

**Table:**

Bruker APEXII CCD diffractometer	2359 independent reflections
Radiation source: fine-focus sealed tube	1930 reflections with *I* > 2σ(*I*)
Graphite monochromator	*R*_int_ = 0.023
Detector resolution: 0.95 pixels mm^-1^	θ_max_ = 22.4°, θ_min_ = 2.6°
φ and ω scans	*h* = −18→15
Absorption correction: multi-scan (*SADABS*; Bruker, 2009)	*k* = −8→7
*T*_min_ = 0.942, *T*_max_ = 0.958	*l* = −16→16
11988 measured reflections	

### Refinement

**Table:**

Refinement on *F*^2^	Primary atom site location: structure-invariant direct methods
Least-squares matrix: full	Secondary atom site location: difference Fourier map
*R*[*F*^2^ > 2σ(*F*^2^)] = 0.033	Hydrogen site location: inferred from neighbouring sites
*wR*(*F*^2^) = 0.092	H-atom parameters constrained
*S* = 1.06	*w* = 1/[σ^2^(*F*_o_^2^) + (0.0494*P*)^2^ + 0.3661*P*] where *P* = (*F*_o_^2^ + 2*F*_c_^2^)/3
2359 reflections	(Δ/σ)_max_ = 0.001
236 parameters	Δρ_max_ = 0.15 e Å^−^^3^
0 restraints	Δρ_min_ = −0.23 e Å^−^^3^
0 constraints	

### Special details

**Table:**

Geometry. All e.s.d.'s (except the e.s.d. in the dihedral angle between two l.s. planes) are estimated using the full covariance matrix. The cell e.s.d.'s are taken into account individually in the estimation of e.s.d.'s in distances, angles and torsion angles; correlations between e.s.d.'s in cell parameters are only used when they are defined by crystal symmetry. An approximate (isotropic) treatment of cell e.s.d.'s is used for estimating e.s.d.'s involving l.s. planes.
Refinement. Refinement of *F*^2^ against ALL reflections. The weighted *R*-factor *wR* and goodness of fit *S* are based on *F*^2^, conventional *R*-factors *R* are based on *F*, with *F* set to zero for negative *F*^2^. The threshold expression of *F*^2^ > σ(*F*^2^) is used only for calculating *R*-factors(gt) *etc*. and is not relevant to the choice of reflections for refinement. *R*-factors based on *F*^2^ are statistically about twice as large as those based on *F*, and *R*- factors based on ALL data will be even larger.

### Fractional atomic coordinates and isotropic or equivalent isotropic displacement parameters (Å^2^)

**Table:**

	*x*	*y*	*z*	*U*_iso_*/*U*_eq_	
C1	0.56842 (15)	0.1855 (3)	−0.19563 (16)	0.0606 (6)	
C2	0.50273 (17)	0.2154 (3)	−0.28076 (15)	0.0652 (6)	
C3	0.42103 (17)	0.2278 (3)	−0.28889 (17)	0.0712 (7)	
H3	0.3767	0.2486	−0.3470	0.085*	
C4	0.40468 (16)	0.2091 (3)	−0.20946 (19)	0.0788 (7)	
H4	0.3488	0.2165	−0.2137	0.095*	
C5	0.47062 (17)	0.1794 (3)	−0.12370 (17)	0.0710 (7)	
H5	0.4587	0.1672	−0.0705	0.085*	
C6	0.55410 (14)	0.1675 (3)	−0.11526 (14)	0.0542 (6)	
C7	0.62575 (15)	0.1198 (3)	−0.02411 (15)	0.0599 (6)	
C8	0.71988 (15)	0.1996 (3)	0.13429 (14)	0.0644 (6)	
H8A	0.7369	0.0777	0.1353	0.077*	
H8B	0.6944	0.2135	0.1797	0.077*	
C9	0.79693 (15)	0.3155 (3)	0.16094 (15)	0.0632 (6)	
H9A	0.8364	0.2901	0.2249	0.076*	
H9B	0.8260	0.2935	0.1195	0.076*	
C10	0.71040 (13)	0.5453 (3)	0.05710 (14)	0.0574 (6)	
H10A	0.7382	0.5278	0.0141	0.069*	
H10B	0.6938	0.6679	0.0542	0.069*	
C11	0.63270 (14)	0.4302 (3)	0.02931 (15)	0.0598 (6)	
H11A	0.6015	0.4580	0.0679	0.072*	
H11B	0.5954	0.4525	−0.0358	0.072*	
C12	0.91357 (14)	0.6645 (3)	0.15849 (14)	0.0584 (6)	
C13	0.98919 (15)	0.5691 (3)	0.19152 (16)	0.0659 (6)	
H13	1.0022	0.4951	0.2431	0.079*	
C14	1.04489 (16)	0.5843 (3)	0.14784 (19)	0.0746 (7)	
H14	1.0952	0.5191	0.1702	0.089*	
C15	1.02791 (17)	0.6943 (3)	0.07146 (18)	0.0730 (7)	
C16	0.95135 (18)	0.7848 (3)	0.03828 (17)	0.0742 (7)	
H16	0.9379	0.8565	−0.0143	0.089*	
C17	0.89459 (16)	0.7722 (3)	0.08056 (16)	0.0659 (6)	
H17	0.8437	0.8354	0.0571	0.079*	
C18	1.0916 (2)	0.7155 (4)	0.0280 (2)	0.1021 (10)	
H18A	1.0634	0.7612	−0.0346	0.153*	
H18B	1.1165	0.6034	0.0256	0.153*	
H18C	1.1357	0.7956	0.0651	0.153*	
N1	0.65731 (11)	0.2441 (2)	0.04104 (11)	0.0572 (5)	
N2	0.76965 (11)	0.5003 (2)	0.15333 (11)	0.0571 (5)	
O1	0.65057 (13)	−0.0330 (2)	−0.01083 (11)	0.0937 (6)	
O2	0.88639 (11)	0.5727 (2)	0.30408 (10)	0.0772 (5)	
O3	0.79753 (10)	0.8085 (2)	0.20235 (11)	0.0740 (5)	
F1	0.64895 (9)	0.1741 (2)	−0.19068 (10)	0.0971 (5)	
F2	0.52079 (11)	0.2303 (2)	−0.35774 (10)	0.1029 (6)	
S1	0.84155 (4)	0.64492 (7)	0.21283 (4)	0.0619 (2)	

### Atomic displacement parameters (Å^2^)

**Table:**

	*U*^11^	*U*^22^	*U*^33^	*U*^12^	*U*^13^	*U*^23^
C1	0.0636 (15)	0.0573 (14)	0.0623 (15)	0.0075 (12)	0.0267 (13)	0.0050 (11)
C2	0.0869 (18)	0.0589 (15)	0.0500 (14)	0.0087 (13)	0.0274 (14)	0.0016 (10)
C3	0.0761 (18)	0.0625 (16)	0.0607 (15)	0.0060 (13)	0.0122 (13)	−0.0038 (11)
C4	0.0635 (16)	0.0821 (19)	0.087 (2)	0.0011 (14)	0.0268 (16)	−0.0009 (14)
C5	0.0809 (18)	0.0705 (16)	0.0687 (16)	0.0017 (14)	0.0372 (15)	0.0020 (12)
C6	0.0702 (15)	0.0380 (12)	0.0526 (13)	0.0016 (10)	0.0226 (11)	−0.0001 (9)
C7	0.0803 (16)	0.0451 (15)	0.0530 (13)	0.0055 (12)	0.0252 (12)	0.0038 (11)
C8	0.0877 (17)	0.0441 (13)	0.0535 (13)	0.0094 (12)	0.0198 (12)	0.0069 (10)
C9	0.0733 (15)	0.0474 (14)	0.0556 (13)	0.0164 (12)	0.0117 (12)	0.0094 (10)
C10	0.0643 (14)	0.0413 (13)	0.0549 (13)	0.0079 (11)	0.0115 (11)	0.0077 (9)
C11	0.0672 (14)	0.0435 (13)	0.0596 (13)	0.0097 (11)	0.0158 (11)	0.0037 (10)
C12	0.0631 (14)	0.0409 (12)	0.0522 (12)	−0.0002 (11)	0.0031 (11)	−0.0037 (10)
C13	0.0656 (15)	0.0497 (14)	0.0621 (14)	0.0027 (12)	0.0040 (13)	−0.0012 (11)
C14	0.0597 (15)	0.0575 (16)	0.0884 (18)	0.0001 (13)	0.0105 (14)	−0.0153 (14)
C15	0.0815 (18)	0.0529 (15)	0.0763 (17)	−0.0159 (14)	0.0227 (15)	−0.0202 (13)
C16	0.0898 (19)	0.0559 (16)	0.0635 (15)	−0.0092 (14)	0.0163 (15)	−0.0017 (12)
C17	0.0702 (15)	0.0513 (15)	0.0592 (14)	0.0028 (12)	0.0079 (13)	0.0031 (11)
C18	0.109 (2)	0.089 (2)	0.120 (2)	−0.0272 (18)	0.058 (2)	−0.0307 (18)
N1	0.0741 (12)	0.0373 (11)	0.0504 (10)	0.0061 (9)	0.0144 (9)	0.0024 (8)
N2	0.0660 (11)	0.0395 (10)	0.0518 (10)	0.0089 (9)	0.0089 (9)	0.0057 (8)
O1	0.1381 (16)	0.0460 (11)	0.0679 (10)	0.0203 (10)	0.0104 (10)	−0.0024 (8)
O2	0.0933 (12)	0.0732 (11)	0.0470 (8)	0.0107 (9)	0.0088 (8)	0.0031 (7)
O3	0.0833 (11)	0.0487 (9)	0.0760 (10)	0.0128 (8)	0.0169 (9)	−0.0088 (7)
F1	0.0793 (10)	0.1325 (14)	0.0878 (10)	0.0192 (9)	0.0422 (8)	0.0291 (9)
F2	0.1248 (13)	0.1301 (14)	0.0601 (9)	0.0342 (11)	0.0437 (9)	0.0201 (8)
S1	0.0722 (4)	0.0485 (4)	0.0499 (3)	0.0083 (3)	0.0084 (3)	−0.0018 (2)

### Geometric parameters (Å, º)

**Table:**

C1—F1	1.347 (2)	C10—H10A	0.9700
C1—C2	1.367 (3)	C10—H10B	0.9700
C1—C6	1.369 (3)	C11—N1	1.466 (3)
C2—C3	1.351 (3)	C11—H11A	0.9700
C2—F2	1.352 (3)	C11—H11B	0.9700
C3—C4	1.375 (3)	C12—C13	1.387 (3)
C3—H3	0.9300	C12—C17	1.387 (3)
C4—C5	1.375 (3)	C12—S1	1.751 (2)
C4—H4	0.9300	C13—C14	1.374 (3)
C5—C6	1.379 (3)	C13—H13	0.9300
C5—H5	0.9300	C14—C15	1.383 (4)
C6—C7	1.498 (3)	C14—H14	0.9300
C7—O1	1.225 (3)	C15—C16	1.380 (4)
C7—N1	1.330 (3)	C15—C18	1.497 (4)
C8—N1	1.454 (3)	C16—C17	1.372 (3)
C8—C9	1.496 (3)	C16—H16	0.9300
C8—H8A	0.9700	C17—H17	0.9300
C8—H8B	0.9700	C18—H18A	0.9600
C9—N2	1.470 (3)	C18—H18B	0.9600
C9—H9A	0.9700	C18—H18C	0.9600
C9—H9B	0.9700	N2—S1	1.6311 (17)
C10—N2	1.470 (2)	O2—S1	1.4234 (15)
C10—C11	1.500 (3)	O3—S1	1.4281 (16)
			
F1—C1—C2	119.3 (2)	C10—C11—H11A	109.5
F1—C1—C6	119.3 (2)	N1—C11—H11B	109.5
C2—C1—C6	121.4 (2)	C10—C11—H11B	109.5
C3—C2—F2	120.1 (2)	H11A—C11—H11B	108.1
C3—C2—C1	121.2 (2)	C13—C12—C17	119.3 (2)
F2—C2—C1	118.7 (2)	C13—C12—S1	120.31 (18)
C2—C3—C4	118.6 (2)	C17—C12—S1	120.34 (18)
C2—C3—H3	120.7	C14—C13—C12	119.8 (2)
C4—C3—H3	120.7	C14—C13—H13	120.1
C3—C4—C5	120.3 (2)	C12—C13—H13	120.1
C3—C4—H4	119.9	C13—C14—C15	121.6 (2)
C5—C4—H4	119.9	C13—C14—H14	119.2
C4—C5—C6	121.1 (2)	C15—C14—H14	119.2
C4—C5—H5	119.4	C16—C15—C14	117.7 (3)
C6—C5—H5	119.4	C16—C15—C18	121.7 (3)
C1—C6—C5	117.3 (2)	C14—C15—C18	120.6 (3)
C1—C6—C7	120.6 (2)	C17—C16—C15	121.9 (2)
C5—C6—C7	121.8 (2)	C17—C16—H16	119.0
O1—C7—N1	122.5 (2)	C15—C16—H16	119.0
O1—C7—C6	119.0 (2)	C16—C17—C12	119.6 (2)
N1—C7—C6	118.36 (19)	C16—C17—H17	120.2
N1—C8—C9	110.67 (17)	C12—C17—H17	120.2
N1—C8—H8A	109.5	C15—C18—H18A	109.5
C9—C8—H8A	109.5	C15—C18—H18B	109.5
N1—C8—H8B	109.5	H18A—C18—H18B	109.5
C9—C8—H8B	109.5	C15—C18—H18C	109.5
H8A—C8—H8B	108.1	H18A—C18—H18C	109.5
N2—C9—C8	109.02 (18)	H18B—C18—H18C	109.5
N2—C9—H9A	109.9	C7—N1—C8	120.31 (17)
C8—C9—H9A	109.9	C7—N1—C11	125.61 (17)
N2—C9—H9B	109.9	C8—N1—C11	114.06 (16)
C8—C9—H9B	109.9	C9—N2—C10	111.79 (16)
H9A—C9—H9B	108.3	C9—N2—S1	117.19 (14)
N2—C10—C11	109.02 (16)	C10—N2—S1	118.30 (13)
N2—C10—H10A	109.9	O2—S1—O3	119.91 (10)
C11—C10—H10A	109.9	O2—S1—N2	106.66 (9)
N2—C10—H10B	109.9	O3—S1—N2	106.31 (9)
C11—C10—H10B	109.9	O2—S1—C12	108.06 (10)
H10A—C10—H10B	108.3	O3—S1—C12	107.97 (10)
N1—C11—C10	110.57 (17)	N2—S1—C12	107.32 (9)
N1—C11—H11A	109.5		
			
F1—C1—C2—C3	180.0 (2)	C15—C16—C17—C12	−0.6 (3)
C6—C1—C2—C3	−0.2 (4)	C13—C12—C17—C16	−1.0 (3)
F1—C1—C2—F2	0.8 (3)	S1—C12—C17—C16	−179.41 (17)
C6—C1—C2—F2	−179.4 (2)	O1—C7—N1—C8	−4.0 (3)
F2—C2—C3—C4	178.9 (2)	C6—C7—N1—C8	172.92 (19)
C1—C2—C3—C4	−0.2 (4)	O1—C7—N1—C11	178.1 (2)
C2—C3—C4—C5	0.4 (4)	C6—C7—N1—C11	−5.1 (3)
C3—C4—C5—C6	−0.1 (4)	C9—C8—N1—C7	127.7 (2)
F1—C1—C6—C5	−179.7 (2)	C9—C8—N1—C11	−54.1 (3)
C2—C1—C6—C5	0.5 (3)	C10—C11—N1—C7	−128.3 (2)
F1—C1—C6—C7	−5.4 (3)	C10—C11—N1—C8	53.6 (2)
C2—C1—C6—C7	174.8 (2)	C8—C9—N2—C10	−60.2 (2)
C4—C5—C6—C1	−0.3 (3)	C8—C9—N2—S1	158.59 (15)
C4—C5—C6—C7	−174.6 (2)	C11—C10—N2—C9	59.8 (2)
C1—C6—C7—O1	−76.6 (3)	C11—C10—N2—S1	−159.43 (14)
C5—C6—C7—O1	97.4 (3)	C9—N2—S1—O2	−44.59 (18)
C1—C6—C7—N1	106.4 (2)	C10—N2—S1—O2	176.77 (15)
C5—C6—C7—N1	−79.5 (3)	C9—N2—S1—O3	−173.63 (15)
N1—C8—C9—N2	55.6 (2)	C10—N2—S1—O3	47.72 (18)
N2—C10—C11—N1	−54.7 (2)	C9—N2—S1—C12	71.02 (17)
C17—C12—C13—C14	1.0 (3)	C10—N2—S1—C12	−67.62 (17)
S1—C12—C13—C14	179.43 (16)	C13—C12—S1—O2	18.83 (19)
C12—C13—C14—C15	0.5 (3)	C17—C12—S1—O2	−162.78 (17)
C13—C14—C15—C16	−2.0 (3)	C13—C12—S1—O3	149.92 (17)
C13—C14—C15—C18	176.9 (2)	C17—C12—S1—O3	−31.70 (19)
C14—C15—C16—C17	2.1 (3)	C13—C12—S1—N2	−95.85 (17)
C18—C15—C16—C17	−176.9 (2)	C17—C12—S1—N2	82.53 (18)

### Hydrogen-bond geometry (Å, º)

**Table:**

*D*—H···*A*	*D*—H	H···*A*	*D*···*A*	*D*—H···*A*
C4—H4···O3^i^	0.93	2.58	3.495 (3)	169
C8—H8*A*···O3^ii^	0.97	2.34	3.255 (3)	157
C10—H10*B*···O1^iii^	0.97	2.48	3.402 (3)	159
C8—H8*B*···F1^iv^	0.97	2.57	3.518 (3)	165
C11—H11*A*···F2^iv^	0.97	2.56	3.296 (3)	133

Symmetry codes: (i) −*x*+1, −*y*+1, −*z*; (ii) *x*, *y*−1, *z*; (iii) *x*, *y*+1, *z*; (iv) *x*, −*y*+1/2, *z*+1/2.
